# Modelling and optimal control of multi strain epidemics, with application to COVID-19

**DOI:** 10.1371/journal.pone.0257512

**Published:** 2021-09-16

**Authors:** Edilson F. Arruda, Shyam S. Das, Claudia M. Dias, Dayse H. Pastore

**Affiliations:** 1 Department of Decision Analytics and Risk, Southampton Business School, University of Southampton, Southampton, United Kingdom; 2 Department of Basic and General Disciplines, Federal Center for Technological Education Celso Suckow da Fonseca, Rio de Janeiro, Rio de Janeiro, Brazil; 3 Graduate Program in Mathematical and Computational Modeling, Multidisciplinary Institute, Federal Rural University of Rio de Janeiro, Nova Iguaçu RJ, Brazil; Centers for Disease Control and Prevention, UNITED STATES

## Abstract

Reinfection and multiple viral strains are among the latest challenges in the current COVID-19 pandemic. In contrast, epidemic models often consider a single strain and perennial immunity. To bridge this gap, we present a new epidemic model that simultaneously considers multiple viral strains and reinfection due to waning immunity. The model is general, applies to any viral disease and includes an optimal control formulation to seek a trade-off between the societal and economic costs of mitigation. We validate the model, with and without mitigation, in the light of the COVID-19 epidemic in England and in the state of Amazonas, Brazil. The model can derive optimal mitigation strategies for any number of viral strains, whilst also evaluating the effect of distinct mitigation costs on the infection levels. The results show that relaxations in the mitigation measures cause a rapid increase in the number of cases, and therefore demand more restrictive measures in the future.

## Introduction

Also known as COVID-19, the Severe Acute Respiratory Syndrome Coronavirus 2 (SARS-CoV-2) is believed to have appeared at the end of 2019 in Wuhan, China [1]. This new, highly transmissible virus spread rapidly around the globe, causing significant loss of life and possibly long-lasting economic consequences. The ensuing pandemic highlighted the need for comprehensive epidemic models to help shape public policy [2]. One important challenge is to reconcile inaccurate data reports and conflicting information from distinct studies [3]. Another challenge is to find general modelling frameworks to address newly discovered characteristics, such as reinfection and multiple viral strains [4–6].

Parsimonious models, such as the classical SEIR (Susceptible, Exposed, Infected, Removed), are invaluable for forecasting epidemic spread and to support decision making [7]. Indeed, SEIR belongs to the class of compartmental models introduced in the first half of the 20th century to describe the spread of transmissible diseases [8, 9]. Simple and easy to use, they were able to predict the spread of COVID-19 in US states [7] and to fit historical data of the 1918 flu epidemic in the US [10]. Works such as [11] proved to be very useful in predicting the spread of COVID-19 in different countries and regions.

Many mathematical models and data analytics tools have been proposed to understand the evolution of the COVID-19 pandemic throughout the world, generally based on the SEIR classical compartmental model (see [12] for an overview of mathematical modelling applications to COVID-19). We found in the literature different models for COVID-19, developed mainly to study the influence of social distancing and non-pharmaceutical interventions on disease progression [13, 14]. The study in [2] promoted non-pharmaceutical interventions, whereas [15] assessed the effect of such measures in Europe. Researchers evaluated the effectiveness of long-term on-off lock-down policies [16], and pursued optimal trade-offs between economics and healthcare concerns [17]. Like most of the literature, these works did not consider the possibility of reinfection or multiple viral strains. Similarly, these possibilities were also disregarded in investigations of optimal strategies to exit lock-down, which also neglected the possibility of multiple waves of infection [18, 19].

To shape public policy, we also need a thorough understanding of the pandemic. This includes mapping the genomics of viral strains [20, 21]. Indeed, researchers recently mapped new COVID-19 strains in the United Kingdom [22] and South Africa [23], which have rapidly spread around the globe. In Brazil, initial studies revealed more than 100 COVID-19 viral strains [6, 24, 25], three of which survived. Such a reduction in genetic diversity has been attributed to the social isolation measures in that country [26]. Recently, a variant known as P.1 (lineage 501Y.V3 or Brazilian variant) has become prevalent in Brazil. Sequencing results from the state of Amazonas, Brazil—where the variant was first detected—identified P.1 in about 42% of the samples tested in December 2020 [27]. This variant is believed to have a high potential for reinfection [6].

Another important challenge to modellers is that the immune response to COVID-19 is not uniform [28], may reportedly wane over time [29–31] and reinfection is possible [4, 5]. Furthermore, the same patient may be infected by different strains of the virus [32, 33]. As stated in [34], a thorough understanding of reinfection is essential for understanding the spread of the disease, as future global challenges include containing epidemics with reinfection [35].

For data-based modelling, we refer the interested reader to [36]. This work utilised available databases and the classical SIR (Susceptible, Infected, Recovered) framework to estimate the number of COVID-19 reinfections from empirical data. Additionally, [37] discusses the challenges of applying data-science to COVID-19, which include reconciling conflicting and inaccurate reports, partly due to asymptomatic infections and insufficient testing.

Although COVID-19 reinfection and multiple viral strains have received increased attention in the literature, mathematical modelling that incorporates these characteristics is still scarce. A general two-strain model searched for stability conditions and assessed a quarantine strategy to curb COVID-19 in Morocco [38]. More generally, viral reinfection is often studied with emphasis on stability conditions and disease-free equilibrium [39]. Specifically, [40] featured a SEIR model for swine influenza and evaluated prescribed vaccination strategies. Finally, a simpler SIR model studied the dynamics of two viral strains, considering that the second strain appears after the first strain reaches equilibrium [41]. In general, whilst these models examine long-term stability, they do not incorporate decision support tools and optimisation.

To support decision making, optimal control approaches have been proposed to promote compromises between COVID-19 infection levels and economic consequences of non-pharmaceutical interventions [17, 42, 43]. The control may comprise a proportional reduction in infection [17, 43] or include quarantine, isolation, and public health education [42]. Although these models do not consider reinfection and multiple viral strains, they do provide interesting insights. An interesting insight is that, to preserve healthcare systems and leverage control options late in the epidemics, we need high levels of control from the outset [43]. This is consistent with the empirical results in [16], which combined the SEIR model with on/off lock-down policies to assess the impact of spreading the outbreak across several waves of decreasing amplitude. The results showed that there would exist multiple waves requiring flattening over time in the absence of effective medication, an appropriate vaccine, or the development of herd immunity.

Whereas models considering multiple viral strains are rare, the literature contains optimal control approaches based on classical epidemiological models for two viral strains [44, 45]. These are general epidemiological models, i.e. not specifically tailored for a given epidemic, that do not consider reinfection. A limiting feature of the model in [44], however, is that it relies on curative treatment. In contrast, the discrete network-based model in [45] relies on separate control measures for each strain.

To the best of our knowledge, this is the first paper to simultaneously consider multiple viral strains, reinfection, and optimal control. Amongst the novel contributions of this work, we generalise the preceding literature [38, 44, 45] by considering not only two but any number of viral strains. Based on the SEIR framework, the model innovates by considering waning immunity over time, as well as reinfection, which can considerably increase the infection levels. Finally, we propose a novel optimal control approach whereby a proportional reduction of the infection rate by mitigation measures (such as non-pharmaceutical interventions) incurs an exponentially increasing cost. This approach is more realistic than assuming linear or quadratic costs [42, 43], once it is increasingly difficult—and therefore costlier—to reduce transmission after mitigating measures are already in place. The proposed approach seeks a compromise between the overall number of deaths and the intervention costs over a prescribed horizon.

In addition to the methodological innovations, we also contribute by providing a more realistic framework for epidemic modelling that avoids the sometimes optimistic assumptions of perennial immunity and a single viral strain. The framework also includes an optimal control formulation that enables decision makers to define a compromise between loss of life and economic consequences over a prolonged time horizon. It is worth emphasising that, although the COVID-19 pandemic is certainly a motivation, we propose a general framework for a realistic modelling of the spread of viral diseases. As such, it includes the possibility of reinfection due to waning immunity, as well as multiple viral strains and optimal control.

The remainder of this paper is organised as follows. We firstly introduce the proposed multi-strain model with reinfection and analyse its equilibrium points and the reproductive number. Then, we propose a novel optimal control formulation for the multi-strain model, which is solved to derive the optimal control strategy over a prescribed time horizon. Next, we propose a series of experiments designed to illustrate the system’s behaviour in the presence of two strains, with and without mitigation. The experiments consider the largely unmitigated COVID-19 spread in the state of Amazonas, Brazil [3, 46, 47], where the epidemic gave rise to two distinct viral strains in 2020 [27]; started in April 2021, the vaccination had no effect on the first two waves. Furthermore, the estimated 75% attack rate during the first wave [46] implies the second peak is mainly due to the second strain. For the unmitigated epidemic, the model’s results are compatible with the observed outbreak and explain the attack rate observed in a serological study. To further validate our model, we apply it to the second and third waves of COVID-19 in England, where distinct mitigation measures were applied. The results are compatible with the infection levels observed in the country. We also derive and interpret optimal control strategies for the Amazonas epidemic, over a two-year horizon and under distinct mitigation costs and two viral strains. Finally, we present our concluding remarks.

### Preliminaries

**Fig 1:**
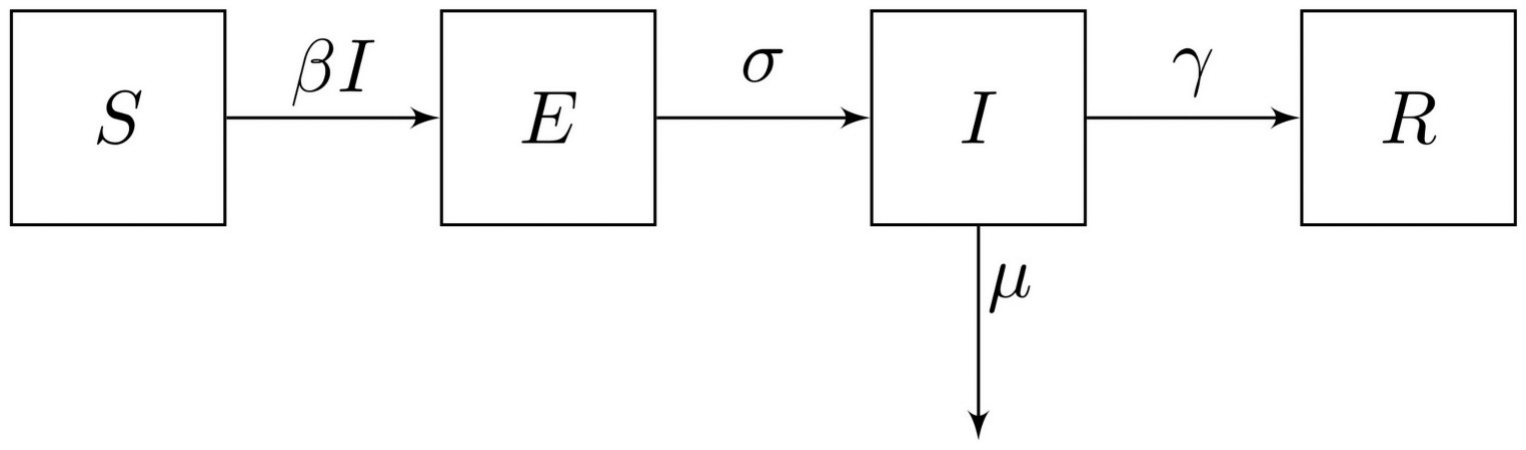
The classical SEIR model.

Introduced in the first half of the 20th century [8], the classical SEIR model divides the population into four compartments: *susceptible* (*S*), exposed (*E*), infected (*I*) and *removed* (*R*). The system’s dynamics follows the equations below and is illustrated in Fig 1, which depicts the transitions among the compartments.

$$\begin{array}{cc} \dot{S}\left(t\right) & =-\beta S(t)I(t)\\ \dot{E}\left(t\right) & =\beta S(t)I(t)-\sigma E(t)\\ \dot{I}\left(t\right) & =\sigma E\left(t\right)-(\;\mu \;+\;\gamma \;)I\left(t\right)\\ \dot{R}\left(t\right) & =\gamma I(t) \end{array}$$

As observed in the equations above and in Fig 1, healthy individuals are *susceptible* to the disease and can acquire it upon encountering *infected* individuals. The total number of encounters is *SI* and the rate of transmission per encounter is *β*. Hence, *susceptible* individuals become *exposed* to the disease at an overall rate *βSI*. *Exposed* individuals have acquired the disease in a latent state; the disease is not manifested nor can be transmitted while the latency period lasts. As the duration of the latency period is $\frac{1}{\sigma }$, *exposed* individuals become *infected* at an overall rate *σE*. The infection lasts $\frac{1}{\gamma }$ units of time, therefore *infected* individuals become *removed* at an overall rate *γI*. Alternatively, *infected* individuals may die, at rate *μ*. Finally, *removed* individuals have acquired immunity through infection and can no longer be affected by the illness.

Observe in Fig 1 that the dynamics of the classical SEIR model imply perennial immunity, as removed individuals can no longer be affected by the disease. Another important characteristic of the model is that it considers a single viral strain. In the next section, we will introduce a generalised model that considers multiple strains, waning immunity and a control variable *u*(*t*) to account for mitigation measures.

## Proposed mathematical model

Let *V* = {1, …, *n*} be the set of virus strains circulating in the population, and let *j* ∈ *V* denote a particular strain. For each *j* ∈ *V* and time *t* ≥ 0, let *S*_*j*_(*t*), *E*_*j*_(*t*), *I*_*j*_(*t*) and *R*_*j*_(*t*) respectively denote the number of susceptible, exposed, infected and removed (recovered and immune) individuals in the population at time *t*. In addition, *P*(*t*) denotes the total population at time *t* ≥ 0.

The susceptible population *S*_*j*_(*t*) includes all individuals that are not immune to strain *j* ∈ *V* at time *t* ≥ 0 and therefore can catch the disease. In turn, *E*_*j*_(*t*) comprises all individuals that have been recently contaminated by strain *j* but are currently in the latency period and therefore have not yet manifested the disease and become infectious. Finally, *I*_*j*_(*t*) counts all individuals that have caught and manifested the strain *j* and are still suffering from it, whereas *R*_*j*_(*t*) denotes the total number of individuals that are recovered and immune to strain *j* at time *t*.

The proposed multi strain model follows Eqs (1)–(5) below:

$$\begin{array}{c} \dot{P}\left(t\right)=-\sum_{j=1}^{n}\mu_{j}I_{j} \end{array}$$ (1)

$$\begin{array}{c} S_{j}\left(t\right)=P\left(t\right)-E_{j}\left(t\right)-I_{j}\left(t\right)-R_{j}\left(t\right) \end{array}$$ (2)

$$\begin{array}{c} \dot{E_{j}}\left(t\right)=\left(\;1-u\left(t\right)\;\right)\beta_{j}S_{j}\left(t\right)I_{j}\left(t\right)-\sigma_{j}E_{j}\left(t\right), \end{array}$$ (3)

$$\begin{array}{c} \dot{I_{j}}\left(t\right)=\sigma_{j}E_{j}\left(t\right)-\left(\mu_{j}+\gamma_{j}\right)I_{j}\left(t\right), \end{array}$$ (4)

$$\begin{array}{c} \dot{R_{j}}\left(t\right)=\gamma_{j}I_{j}\left(t\right)-\delta_{j}R_{j}\left(t\right), \end{array}$$ (5)

where *S*_*j*_(0) ≤ *P*(0), ∀*j* ∈ *V*.

Consider the dynamics of a given strain *j* ∈ *V*. Observe from Eq (3) that susceptible individuals can contract this strain when in contact with a contagious carrier belonging to the infected population. The rate of infection is *β*_*j*_ > 0 and *u*(*t*) ∈ [0, 1] emulates the mitigation effect at time *t* ≥ 0: *u*(*t*) = 1 indicates 100% effective mitigating measures and *u*(*t*) = 0 represents the absence of non-pharmaceutical interventions, whereas *u*(*t*) ∈ (0,1) indicates partially effective measures to limit the spread of the disease. The first term in the right hand side of (3) represents the formerly susceptible individuals that have just been infected, whereas the second term indicates the exposed individuals that have just manifested the once latent disease. The latter enter the infected compartment in the right hand side of Eq (4). The second term in the right hand side of (4) represents infected individuals that recover—at rate *γ*_*j*_ > 0, or die—at rate *μ*_*j*_ ≥ 0. Finally, each newly recovered individual moves to the *removed* compartment—first term of the right hand side of (5). The second term in the right hand side of (5) represents the loss of immunity over time. Finally, Eq (2) keeps track of the individuals that are currently susceptible to strain *j* ∈ *V*, whereas Eq (1) monitors the evolution of the total population over time. Table 1 describes the system’s parameters.

**Table 1:** Parameters for multi-strain dynamics.

Parameter	Description	Unit
*β*_*j*_	Transmission rate of strain *j*	transmissions/encounter
*σ*_*j*_	Inverse of the latency period of strain *j*	days^−1^
*γ*_*j*_	Recovery rate for strain *j*	days^−1^
*δ*_*j*_	Rate of immunity loss for strain *j*	days^−1^
*μ*_*j*_	Death rate due to strain *j*	days^−1^
*u*(*t*)	Mitigation (lock-down) effect at time *t*	-

**Remark 1***Observe in the system* (1)–(5), *that an individual is susceptible to all strains j* ∈ *V*. *For a particular strain j* ∈ *V*, Eq (2) *ensures that only the individuals that are currently exposed to or infected with strain j are left out of the susceptible population for that strain* (*S*_*j*_), *as well as those currently in the removed compartment of that strain* (*R*_*j*_). *The latter have been recently infected with this strain and are currently immune to it. It is worth reinforcing that this immunity wanes over time at a rate δ*_*j*_—Eq (5).

**Remark 2***A key innovation of the model is to consider multiple viral strains. Observe that the system of* Eqs (1)–(5) *includes an arbitrary number* (*n*) *of viral strains. Another innovation is the possibility of reinfection due to waning immunity, which is contemplated in the last term of* Eq (5). *Therefore, at each time t*, *a fraction δ*_*j*_ *R*_*j*_ *of the individuals currently immune to strain j become susceptible again to this strain and join the susceptible population S*_*j*_: *as R*_*j*_ *decreases*, *S*_*j*_ *increases by the same amount in* Eq (2).

## The equilibrium points

To simplify our analysis, in this section we assume a constant control, i.e. *u*(*t*) = *u* ∈ [0, 1], ∀*t* ≥ 0. A simple inspection to the system of Eqs (1)–(5) yields

$$\begin{array}{c} \dot{S_{j}}\left(t\right)=-\left(\;1-u\;\right)\beta_{j}S_{j}\left(t\right)I_{j}\left(t\right)+\delta_{j}R_{j}\left(t\right)-\sum_{i=1;i\neq j}^{n}\mu_{i}I_{i}\left(t\right). \end{array}$$ (6)

Hence, it is not hard to verify that the trivial equilibrium point is the infection free point, with

$$\begin{array}{c} E_{j}\left(\infty \right)=I_{j}\left(\infty \right)=R_{j}\left(\infty \right)=0,\;S_{j}\left(\infty \right)={\bar{S}}_{j}\geq 0,\;P\left(\infty \right)\geq 0. \end{array}$$ (7)

To calculate the non-trivial equilibrium, we start with the case of two strains below.

**Theorem 1***Suppose that there are n* = 2 *viral strains. Then, besides the trivial equilibrium point in* Eq (7), *the system has a non-trivial equilibrium point with I*_1_ ≤ 0.

From Theorem 1, it follows that the non-trivial equilibrium point of a two-strain model is biologically infeasible, and therefore of no practical interest. Theorem 2 below generalises this result for multiple strains, i.e. |*V*| > 2.

**Theorem 2***Suppose that n* > 2. *Then*, *besides the trivial equilibrium point in* Eq (7), *the system has a non-trivial equilibrium point with I*_*j*_ ≤ 0, ∀*j* ∈ {1, …, *n*}.

Theorem 2 therefore implies that the non-trivial equilibrium point is biologically infeasible and of no practical use for any number of different strains. In the remainder of this paper, we will only consider biologically feasible solutions. The proofs of Theorems 1 and 2 can be found in Appendix A of S1 Appendix.

### Stability

Considering that only the trivial equilibrium points are of biological interest, this section analyses the stability solely with respect to these points.

To prove stability we need to show that the real part of the eigenvalues Jacobian matrix associated with the system and applied to the trivial equilibrium are negative. From the conditions of stability we can define the reproduction number (see the proof in the Appendix A of S1 Appendix),

$$\begin{array}{c} R_{0}=\max_{j=1,...,n}\frac{\left(\;1-u\;\right)\;\beta_{j}\;{\bar{S}}_{j}}{\mu_{j}+\gamma_{j}}. \end{array}$$ (8)

We can say that the trivial equilibrium point (without infection) is locally asymptotically stable if *R*_0_ < 1. Hence, Eq (8) implies a minimum level of constant lock-down effect *u* ∈ [0, 1] to stabilise the system. Observe that, since the lock-down effect applies to all viral strains, it suffices to stabilise the system with respect to the most transmissible strain.

In the next section, we expand the analysis to search for time varying lock-down effects with a view to optimising the long-term cost of non-pharmaceutical (lock-down) interventions.

## Optimal mitigation strategies

To control the spread of the disease in the population, the proposed strategy considers an isolation level of the population *u*(*t*), *t* ≥ 0 at any time *t*. To account for the time-varying control, let us rewrite the system of Eqs (1)–(6) as follows:

$$\begin{array}{c} \dot{P}\left(t\right)=-\sum_{j=1}^{n}\mu_{j}I_{j} \end{array}$$ (9)

$$\begin{array}{c} \dot{S_{j}}\left(t\right)=-\left(1-u\left(t\right)\right)\beta_{j}S_{j}\left(t\right)I_{j}\left(t\right)+\delta_{j}R_{j}\left(t\right)-\sum_{i=1;i\neq j}^{n}\mu_{i}I_{i} \end{array}$$ (10)

$$\begin{array}{c} \dot{E_{j}}\left(t\right)=\left(1-u\left(t\right)\right)\beta_{j}S_{j}\left(t\right)I_{j}\left(t\right)-\sigma_{j}E_{j}\left(t\right), \end{array}$$ (11)

$$\begin{array}{c} \dot{I_{j}}\left(t\right)=\sigma_{j}E_{j}\left(t\right)-\left(\mu_{j}+\gamma_{j}\right)I_{j}\left(t\right), \end{array}$$ (12)

$$\begin{array}{c} \dot{R_{j}}\left(t\right)=\gamma_{j}I_{j}\left(t\right)-\delta_{j}R_{j}\left(t\right), \end{array}$$ (13)

To find a meaningful trade-off between the cost of the control, i.e. lock-down measures or non-pharmaceutical interventions, and the cost of elevated infection levels to the healthcare system and the population in general, we define the following functional cost:

$$\begin{array}{c} J\left(P,u\right)=c_{1}P-e^{c_{2}u},\;0\leq u\leq 1, \end{array}$$ (14)

where *c*_1_ > 0 and *c*_2_ > 0 are scalar parameters.

Recall that in the revised formulation Eqs (9)–(13), *u*(*t*) = 0 indicates no lock-down and *u*(*t*) = 1 corresponds to full lock-down. Observe that the cost in (14) grows with the population size and decreases as a function of the control *u*. While increasing *u* decreases the functional, it also implies a decrease in the number of infections and, therefore, deaths. And less deaths imply an increased total population, thus increasing the functional. Observe also that the cost of control increases exponentially in the feasible interval [0, 1], to mimic the fact that extra mitigation measures tend to become increasingly costly.

Let *ψ* = {*u*(*t*), *t* ∈ (0, *T*): *u*(*t*) ∈ [0, 1]} be a feasible lockdown strategy and let *Ψ* denote the set of all feasible strategies. For each control strategy *ψ* ∈ *Ψ*, let

$$\begin{array}{c} J\left(\psi \right)=\int_{0}^{T}J\left(P\left(s\right),u\left(s\right)\right)\;ds \end{array}$$ (15)

denote the overall cost of the strategy. The optimal control problem then becomes:

$$\begin{array}{c} \begin{array}{c} \text{Maximise}\;J(\psi ),\;\psi \in \Psi \\ \text{subject}\;\text{to}\;(9)-(13). \end{array} \end{array}$$ (16)

The overall objective in (16) is to minimise the number of deaths over time, which is equivalent to maximising the population, whilst also accounting for the cost of lock-down measures represented by the negative term in (14). Theorem 3, in Appendix B of S1 Appendix, guarantees that an optimal solution exists which satisfies (16), and derives the optimal mitigation strategy.

## Numerical experiments

**Fig 2:**
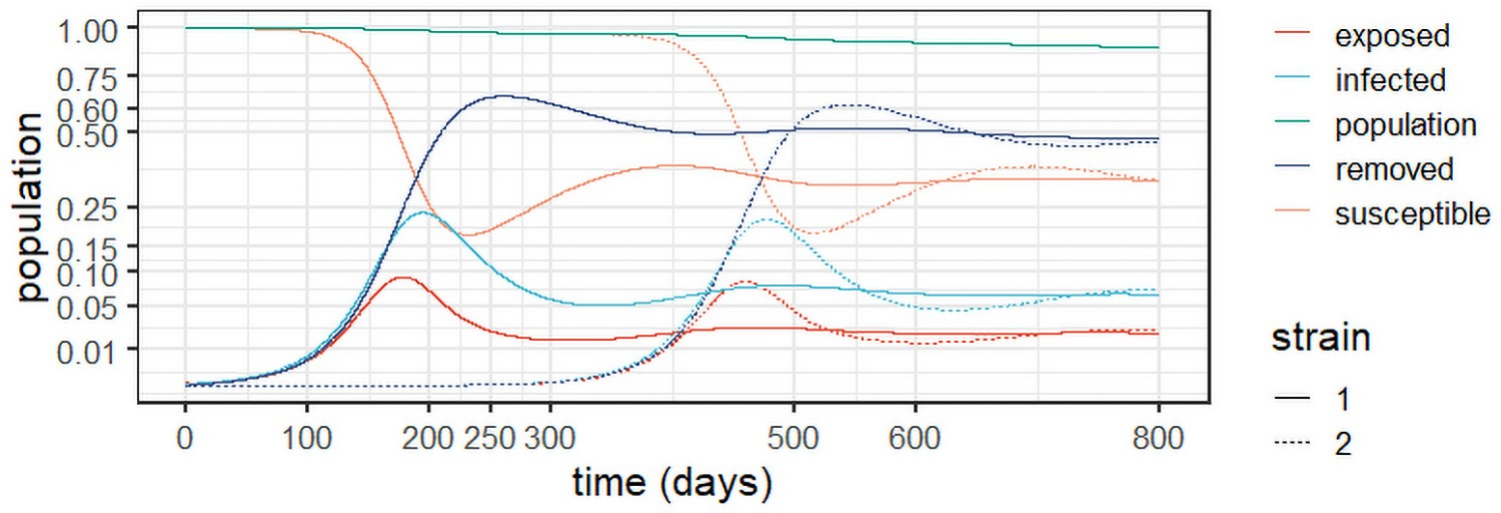
Dynamic behaviour for two strains in Amazonas, Brazil (*Experiment 1*).

To better understand the long-term behaviour of the system (1)–(5), we performed an experiment -termed *Experiment 1*—based on the outbreak at the state of Amazonas in Brazil, an example with reinfection and two viral strains [3]. The experiments used *R*_0_ = 3 as estimated for the state, which yields a 67% overall infection rate [47], just short of the estimate of 76% from a serological study [46]; as, according to [3], this estimate may have been biased due to an adjustment of the observed prevalence of 52.5% due to waning immunity. A second strain called P.1 was detected in the state in December 2020 [27]. Fig 2 depicts the results of *Experiment 1* and Table 2 conveys the model parameters and initial conditions.

**Table 2:** Parameters for *Experiment 1*.

Parameter	Value
*β*_1_ = *β*_2_	3.447 ⋅ 10^−8^
*σ*_1_ = *σ*_2_	$\frac{1}{7}\;{\text{days}}^{-1}$
*γ*_1_ = *γ*_2_	$\frac{1}{21}\;{\text{days}}^{-1}$
*δ*_1_ = *δ*_2_	$\frac{1}{150}\;{\text{days}}^{-1}$
*μ*_1_ = *μ*_2_	1.152 ⋅ 10^−5^days^−1^
*u*(*t*)	0—no mitigation.
**Initial Conditions**	
Strain 1	Strain 2
*S*_1_(0) = 4,144,342	*S*_2_(*t*) = 4,144,597, *t* < 180
*E*_1_(0) = 252	*E*_2_(*t*) = 0,∀*t* ≤ 180,
*I*_1_(0) = 2	*I*_2_(180) = 1, *I*_2_(*t*) = 0,∀*t* < 180
*R*_1_(0) = 1	*R*_2_(*t*) = 0,∀*t* ≤ 180

As the epidemic in Amazonas was largely unmitigated, with a high seroprevalence at the second peak [3, 46], it is consistent with a two-strain outbreak with reinfection, as demonstrated in Fig 2. The stability observed after the first wave can be explained by reinfection from the first strain, whereas at the peak of the second strain more cases are observed as they include patients with both strains. In the experiment, we assumed that the second strain commenced six months (180 days) after the epidemic’s outset. It is worth of emphasis that the removed population stabilises around 50%, in line with the antibody prevalence of 52.5% observed in [46].

Considering that the two strains are similar, the result in Fig 2 is intuitive. We observe that the second strain is simply a delayed version of the first outbreak, which makes sense given the similar parameters. The important feature here is that the second strain will add to the burden on the healthcare system, generating a second peak, increasing the levels of contamination and eventually doubling the burden. Notice, however, that at the peak of the second strain, most of the infections will be from this strain before the system eventually stabilises. Observe also the reduction of the population, which significantly increases after the second strain, as we accumulate deaths from both viral variants. We argue that this should be considered to inform the decision makers. Indeed, strategies to prevent different strains from entering a given territory by enforcing testing upon arrival can be an important part of mitigation policies.

The results corroborate those found in a genetic study in the state of Amazonas, from March 2020 to January 2021 [48]. The study reveals the prevalence of three correlated viral lineages (B.1.1.95, B1.1.28 and B.1.1.33) up to the emergence of variant P.1. Whilst reinfection due to the persistence of the first lineages was the motor of the sustained infection levels up to December 2020, it was the genetically diverse variant P.1 that drove the second wave that started in December 2020.

### An example with control: The case of England

**Fig 3:**
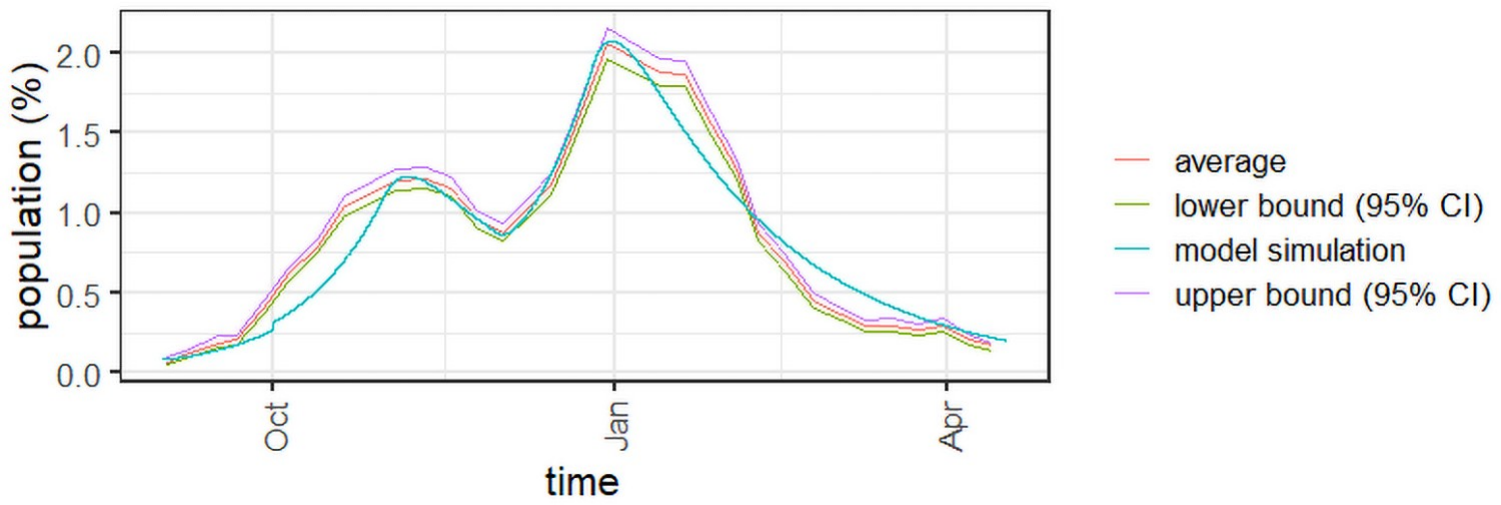
Infected population for Experiment 1a.

To further validate the proposed approach, *Experiment 1a* is based on the second and third COVID-19 waves in England, from September 2020 to April 2021. Variant *Alpha* was first detected in the end of September (https://www.gov.uk/government/publications/covid-19-variants-genomically-confirmed-case-numbers/variants-distribution-of-cases-data). To calibrate the model, we used the results of the weekly survey conducted in England since mid-2020 (https://www.ons.gov.uk/peoplepopulationandcommunity/healthandsocialcare/conditionsanddiseases/bulletins/coronaviruscovid19infectionsurveypilot/previousReleases). The survey provides weekly estimates of the COVID-19 infection levels in the country, as well as a 95% confidence interval, which is depicted in Fig 3 for the selected period. Table 3 features the parameters and initial conditions of the experiment. The R [49] code used to simulate the example is available as supplementary material.

**Table 3:** Parameters for *Experiment 1a*.

Parameter	Value
*β*_1_ = *β*_2_	2.55 ⋅ 10^−9^
*σ*_1_ = *σ*_2_	$\frac{1}{7}\;{\text{days}}^{-1}$
*γ*_1_ = *γ*_2_	$\frac{1}{21}\;{\text{days}}^{-1}$
*δ*_1_ = *δ*_2_	$\frac{1}{150}\;{\text{days}}^{-1}$
*μ*_1_ = *μ*_2_	1.152 ⋅ 10^−5^days^−1^
*u*(*t*)	$\left\{ \begin{array}{cc} 0.18, & 0<t<60\\ 0.82, & 60\leq t<90\\ 0.20, & 90\leq t<116\\ 0.85, & t\geq 116 \end{array}\right.$
**Initial Conditions**	
Strain 1	Strain 2
*S*_1_(0) = 55,932,799	*S*_2_(*t*) = 56,000,000, *t* < 30
*E*_1_(0) = 16,800	*E*_2_(30) = 11200, *E*_2_(*t*) = 0,∀*t* ≤ 180
*I*_1_(0) = 50,400	*I*_2_(30) = 28000, *I*_2_(*t*) = 0,∀*t* < 30
*R*_1_(0) = 1	*R*_2_(*t*) = 0,∀*t* ≤ 30

Observe in Fig 3 that the overall number of infections from the model is consistent with the infection levels observed in the COVID-19 survey in England. The model is able to follow the estimated number of infections whilst also accounting for the varying mitigation measures observed within the time horizon. The values of *u*(*t*) in Table 3 can be seen as estimates of the overall effect of the mitigation measures in place. The larger values correspond to the two lock-down periods observed from September 2020 to April 2021: a one-month lock-down in October and another lock-down period starting in late December, which was still in place in April 2021.

**Fig 4:**
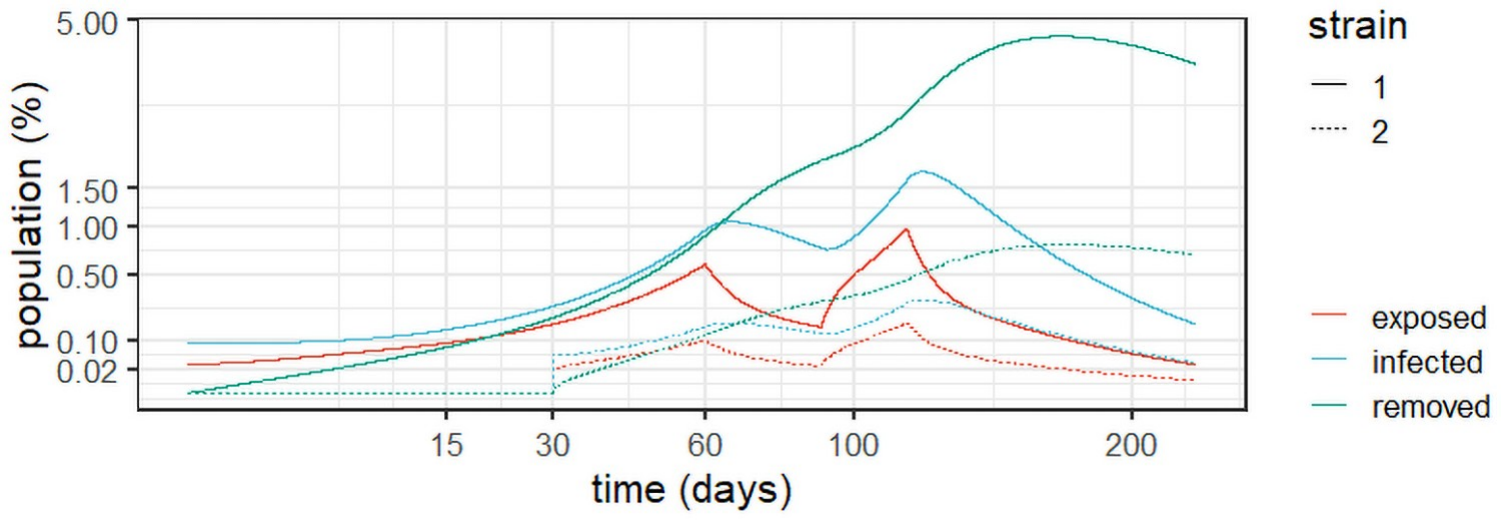
Dynamic behaviour for two strains in England (*Experiment 1a*).

For the sake of completeness, Fig 4 details the evolution of the exposed, infected, and removed populations for *Experiment 1a*. As expected, one can observe two steep increases between lockdown periods and two periods of steady decrease as the lock-downs were put in place.

### Optimal mitigation strategies

**Fig 5:**
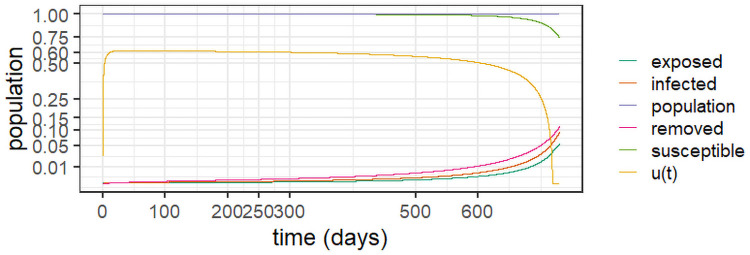
Optimal control policy from the epidemic’s outset (*Experiment 2*), with *c*_1_ = 1 and $c_{2}=\frac{\text{ln}\;(P(0))}{2}$.

This section provides insights into the effect of the optimal control policy derived in Theorem 3 into the dynamics of the system over a two-year horizon. The first simulation is *Experiment 2*, which introduces optimal control at the outset of the epidemic; the parameters of the first strain appear in Table 2. For this experiment, we use *c*_1_ = 1 and $c_{2}=\frac{\text{ln}\;(P(0))}{2}$ in the functional in Eq (14). Depicted in Fig 5, the results show that the optimal control prevents about 60% of the contacts in the early stages and slowly decreases with time. It curbs the epidemic from the outset and therefore inhibits the emergence of the second strain.

**Fig 6:**
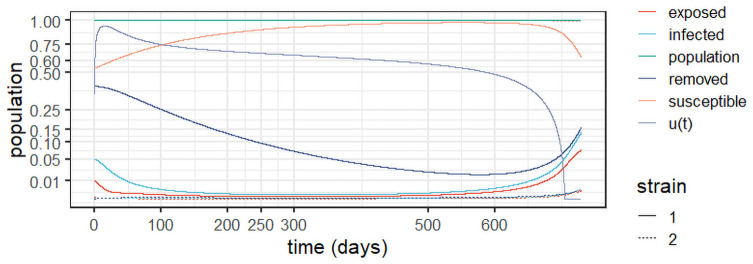
Optimal control policy and system evolution for *Experiment 3*, with *c*_1_ = 1 and $c_{2}=\frac{\text{ln}\;(P(0))}{2}$.

*Experiment 3* assumes stabilisation of the first strain around the values observed in *Experiment 1*, as well as the emergence of a second strain. In other words, this simulates a delayed mitigation policy that starts shortly after the emergence of strain 2. We use the parameters from Table 2 and the initial conditions in Table 4, and make *c*_1_ = 1 and $c_{2}=\frac{\text{ln}\;(P(0))}{2}$ in Eq (14). Observe in Fig 6 that the optimal control starts close to one (full lockdown) to stabilise the first strain; it is continuously reduced over time as the epidemic is effectively mitigated. It is also noteworthy that the control dissipates strain 2 from the outset, as it starts with lower infection levels. Note also that the population remains close to the original levels, indicating the optimal policy’s effective prevention of deaths with respect to the unmitigated scenario in *Experiment 1*.

**Table 4:** Initial conditions for *Experiment 3*.

Initial Conditions	
*S*_1_(0) = 2,238,082	*S*_2_(0) = 4,144,342
*E*_1_(0) = 41446	*E*_2_(0) = 252
*I*_1_(0) = 207230	*I*_2_(0) = 2
*R*_1_(0) = 1657839	*R*_2_(0) = 1

#### Varying parameter *c*_1_ and *c*_2_

We now consider the effects varying parameters *c*_1_ and *c*_2_ in the following experiments, under the same initial conditions and parameters in *Experiment 3*. The cost parameters tested appear in Table 5.

**Table 5:** Cost parameters *c*_1_ and *c*_2_ to be evaluated.

Case	A	B	C	D	E
*c*_1_	1	2	1	3	3
*c*_2_	ln(*P*(0))	ln(*P*(0))	$\frac{\text{ln}(\;P(0)\;)}{3}$	ln(*P*(0))	$\frac{\text{ln}(\;P(0)\;)}{3}$

**Fig 7:**
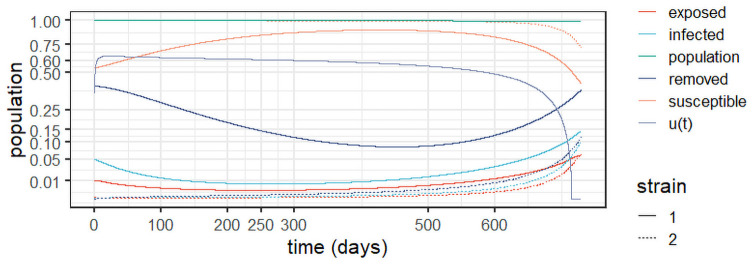
Results for Case A, with *c*_1_ = 1 and *c*_2_ = ln(*P*(0)).

Fig 7 depicts the results for Case A in Table 5. As expected, doubling the cost of control (*c*_2_) with respect to *Experiment 3* in (Fig 6) results in a decrease of the control levels, which start at around 0.6 and slowly decrease over time. That results in a slower stabilisation of the first strain, with higher levels of infection over time. Nonetheless, the control suffices to curb the epidemic and prevent the spread of the second strain, which remains under control over the entire horizon.

**Fig 8:**
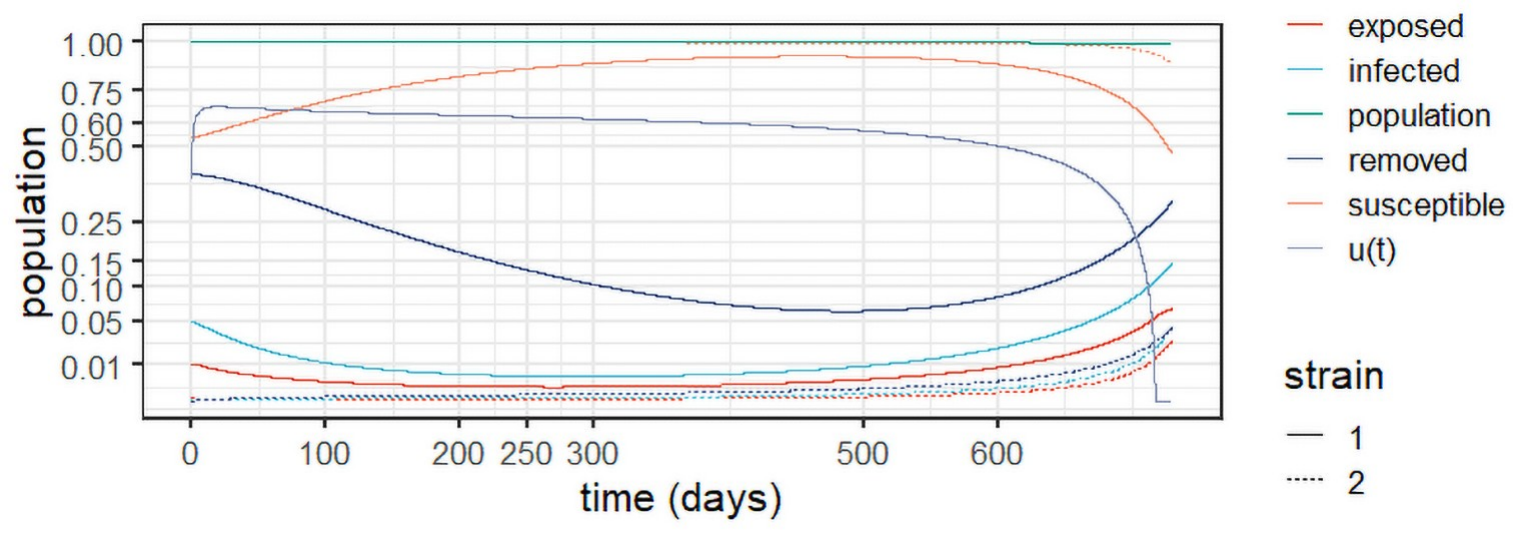
Results for Case B, with *c*_1_ = 2 and *c*_2_ = ln(*P*(0)).

Fig 8 depicts the results for Case B. It uncovers the effect of doubling *c*_1_ with respect to case A, maintaining the same value for *c*_2_. We observe a slight increase in the control levels, as the relative importance of the control costs is decreased. The increase results in slightly lower levels of infections over time for strain 1. The susceptible population therefore increases slightly with respect to Case A, whereas the removed population slightly decreases. As in Case A, strain 2 remains under control for the entire horizon.

**Fig 9:**
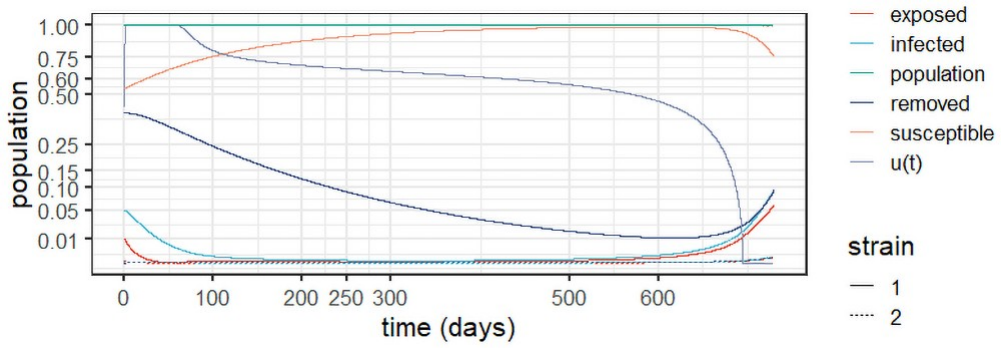
Results for Case C, with *c*_1_ = 1 and $c_{2}=\frac{\text{ln}\;(P(0))}{3}$.

Fig 9 depicts the results for Case C. It uncovers the effect of reducing *c*_2_ to a third of that in Case A, maintaining the same value for *c*_1_. We observe maximum control levels (i.e. full lock-down) for about 60 days to contain the first strain more rapidly, as the relative cost of control decreased. The control is then gradually relaxed as time elapses. The increased control levels result in the near extinction of both strains after about 125 days. Mitigation is then maintained to avoid a resurgence of the disease, as even small levels of infection can lead to another wave. In practice, policy makers may choose more targeted approaches after the infection levels reach a sufficiently small threshold. In that case, the optimal policy will provide guidance as to the desired mitigating effect of such measures to prevent an additional outbreak.

**Fig 10:**
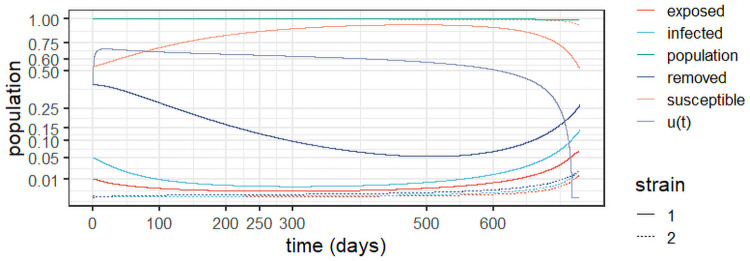
Results for Case D, with *c*_1_ = 3 and *c*_2_ = ln(*P*(0)).

Case D sees *c*_1_ triple with respect to Case A, maintaining the same value of *c*_2_. The results in Fig 10 see a slight increase in control with respect to Cases A and B, with a corresponding slight decrease in infection levels. Therefore, the susceptible population experiences a slight growth with respect to Cases A and B, whereas the removed population slightly falls. As before, strain 2 remains controlled over the entire horizon.

**Fig 11:**
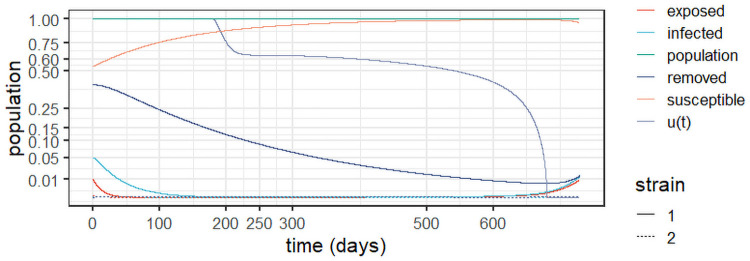
Results for Case E, with *c*_1_ = 3 and $c_{2}=\frac{\text{ln}\;(P(0))}{3}$.

Finally, Case E triples *c*_1_ with respect to Case A while reducing *c*_2_ by two thirds. As depicted in Fig 11, this results in a maximum level of control until both strains are virtually extinguished. Then, the control is quickly reduced to about 0.6 and from there it is slowly reduced to prevent a resurgence. With respect to Case C, we notice a longer time in full lockdown, as the cost of control is decreased, although both strategies quickly extinguish the disease. Once again, the optimal control levels after stabilisation can guide decision makers as to the desired level of mitigation of possibly more targeted prevention policies to prevent resurgence after the epidemic is controlled.

The experiments show that we can derive an optimal control policy to control the outbreak whilst considering both reinfection and multiple strains. *Experiment 2* in Fig 5 suggests that applying an optimal control approach from the outset curbs the epidemic early and hinders the emergency of additional strains. However, the current pandemic vividly reminded us that policy makers may be slow to act and that may result in the appearance of multiple strains. The proposed model provides a general framework to tackle multiple strains and reinfection. *Experiment 3* and Cases A to E demonstrate the potential of the framework to support decision making under a more general setting with multiple strains and reinfections. It allows us to derive an optimal policy to mitigate the epidemic considering the spread of each strain and a prescribed trade-off between societal and economic factors, represented here by cost parameters *c*_1_ and *c*_2_. Cases A to E illustrate how changes in the trade-off will affect the optimal mitigation levels, and consequently the infection levels over time; they also illustrate the need for a proactive policy to prevent the resurgence of the disease after the infection levels are controlled.

### Limitations and future research

The proposed model is devised for emerging epidemics, hence one limitation is that it does not include vaccination. Even though one cannot count on the possibility of quickly developing a vaccine for an emerging epidemic, this possibility would include another level of generality to the model and should be considered in future research.

Another limitation that should be addressed in future research is the fact that the model does not consider cross-immunity between pairs of viral strains. This limitation did not influence our experiments, as the results are compatible with viral strains with no cross-immunity. Indeed, the steep increase in the infection levels in England and in Amazonas suggests very little or no cross-immunity, as confirmed by the experiments. However, although incorporating cross-immunity is not a trivial task, future studies should consider this possibility and suggest ways to include it in the mathematical model.

## Concluding remarks

This paper proposed a novel modelling framework based on the classical SEIR model that considers multiple viral strains, reinfections, and optimal control. Whilst general and applicable to any viral epidemic, the framework was validated in light of the current COVID-19 pandemic, which has challenged healthcare systems around the globe. We applied the approach to the outbreak in the state of Amazonas, Brazil and showed that the outbreak is consistent with a two-strain epidemic with reinfection. The results are interpretable, robust and highlight the applicability of the model to contain viral outbreaks whilst considering the spread of multiple strains and establishing a trade-off between societal and economic impacts.

The results show that, with waning immunity and in the absence of mitigating measures, each viral strain will reach an equilibrium after the peak of infections. Whilst real-world data suggest that the peak is not manageable by any healthcare system in the world, it is evident that even the equilibrium may imply levels of infection that will challenge healthcare resources in many regions of the world. The results also suggest that, with insufficient mitigation measures, an epidemic with a second wave includes a second peak of infections that is higher than the first as it accumulates infections from both strains. Moreover, the number of deaths increases considerably after the emergence of the second strain.

Finally, we proposed and solved an optimal control problem to derive optimal mitigation measures whilst considering that the cost of mitigation grows exponentially as a function of the mitigation effort. Our simulations show that controlling the epidemic from the outset will quickly curb the outbreak, thereby hindering the emergence of different strains and avoiding the devastating effects of a prolonged epidemic. However, the current pandemic has shown that delayed and inadequate mitigation can lead to multiple strains and reinfection. This renders multi-strain models with reinfection invaluable to support decision making in real-world situations, where there is no guarantee that an epidemic will subside before multiple strains appear or that immunity will not wane over time.

We tested our model considering the absence of effective mitigation until the first strain stabilises, exploring the real-world case of the COVID-19 outbreak in the state of Amazonas, Brasil. We found that, when optimal control is activate just after the second strain emerges, it will stabilise the first strain while preventing an outbreak of the second strain. The long-term levels of the disease, as well as the magnitude of the mitigation effort will depend upon the perceived trade-off between societal and economic impacts, in the form of the parameters of the cost functional.

## Supporting information

S1 Appendix (PDF)

S1 File (ZIP)
