# Supplementary material for: Modelling and optimal control of multi strain epidemics, with application to COVID-19
Source: PLoS One. 2021 Sep 16;16(9):e0257512. doi: 10.1371/journal.pone.0257512 (PMC8445490; doi:10.1371/journal.pone.0257512)
Supplement: S1 Appendix — (PDF) [file pone.0257512.s001.pdf]

A Supplementary Material

Proof Theorem 1 Since $|V| = 2$, we have exactly two roots, one of which is the trivial equilibrium. For the non-trivial equilibrium, we must have $I_1 \neq 0$ and $I_2 \neq 0$. Hence, by equalling the left-hand side of (1)-(5) to zero, we obtain:

$$S_1(\infty) = \overline{S}_1 = \frac{\mu_1 + \gamma_1}{(1-u)\beta_1}, \quad S_1(\infty) = \overline{S}_2 = \frac{\mu_2 + \gamma_2}{(1-u)\beta_2}$$

$$E_1(\infty) = \overline{E}_1 = -\frac{(\mu_1 + \gamma_1)\mu_2 \overline{I}_2}{\mu_1 \sigma_1}, \quad E_2(\infty) = \overline{E}_2 = \frac{(\mu_2 + \gamma_2)\overline{I}_2}{\sigma_2},$$

$$I_1(\infty) = \overline{I}_1 = -\frac{\mu_2 \overline{I}_2}{\mu_1}, \quad I_2(\infty) = \overline{I}_2,$$

$$R_1(\infty) = \overline{R}_1 = -\frac{\gamma_1 \mu_2 \overline{I}_2}{\mu_1 \delta_1}, \quad R_2(\infty) = \overline{R}_2 = \frac{\gamma_2 \overline{I}_2}{\delta_2}$$

Since all coefficients are positive, it follows that $\overline{I}_1 \leq 0$.

Proof Theorem 2 Making the left hand side of Eq. (4) equal to zero yields $E_j = \frac{\mu_j+\gamma_j}{\sigma_j} I_j$. Replacing this result in Eq. (3), we obtain:

$$0 = (1-u)\beta_j S_j I_j - \sigma_j E_j = (1-u)\beta_j S_j I_j - (\mu_j + \gamma_j) I_j$$
$$0 = I_j \left((1-u)\beta_j S_j - (\mu_j + \gamma_j) \right).$$

Therefore, either $I_j = 0$ or $S_j = \frac{\mu_j+\gamma_j}{(1-u)\beta_j}$. Now, substituting the latter equality in (6) and making the derivative nil, we have:

$$\begin{aligned}
0 &= -(1-u)\beta_j S_j I_j + \delta_j R_j - \sum_{i=1; i\neq j}^{n} \mu_i I_i \\
&= -(1-u)\beta_j \frac{\mu_j + \gamma_j}{(1-u)\beta_j} I_j + \delta_j R_j - \sum_{i=1; i\neq j}^{n} \mu_i I_i \\
&\implies 0 = -(\mu_j + \gamma_j) I_j + \delta_j R_j - \sum_{i=1; i\neq j}^{n} \mu_i I_i \\
&\implies 0 = -\gamma_j I_j + \delta_j R_j - \sum_{i=1}^{n} \mu_i I_i \\
&\text{eq. (5)} \implies 0 = \sum_{i=1}^{n} \mu_i I_i.
\end{aligned}$$

Since $\mu_j > 0 \ \forall j$, for the last equality to hold we must have either $I_j = 0$, $j = 0, 1 \ \ldots n$, or $I_j < 0$ for at least one $j \in \{1, \ldots, n\}$.

Proof of stability The system (1)-(5) has a dimension $4 \times n + 1$. Consequently, the Jacobian matrix associated with the system and applied to the trivial equilibrium is of order $(4 \times n + 1)^2$. It has $n + 1$ null eigenvalues and n eigenvalues equal to $-\delta_j, j = 1, ..., n$. The remaining $2 \times n$ eigenvalues are given by:

$$-1/2(\mu_j + \gamma_j + \sigma_j) + 1/2 \sqrt{4(1-u)\beta_j \sigma_j \overline{S}_j + (\mu_j + \gamma_j - \sigma_j)^2},$$
$$-1/2(\mu_j + \gamma_j + \sigma_j) - 1/2 \sqrt{4(1-u)\beta_j \sigma_j \overline{S}_j + (\mu_j + \gamma_j - \sigma_j)^2}.$$

Therefore, it suffices to show that

$$-1/2(\mu_j + \gamma_j + \sigma_j) + 1/2 \sqrt{4(1-u)\beta_j \sigma_j \overline{S}_j + (\mu_j + \gamma_j - \sigma_j)^2} < 0,$$

since this implies that the remaining eigenvalues will also be negative. The expression above holds if:

$$\sqrt{4(1-u)\beta_j \sigma_j \overline{S}_j + (\mu_j + \gamma_j - \sigma_j)^2} < (\mu_j + \gamma_j + \sigma_j),$$

$$4(1-u)\beta_j \sigma_j \overline{S}_j + (\mu_j + \gamma_j - \sigma_j)^2 < (\mu_j + \gamma_j + \sigma_j)^2,$$
$$4(1-u)\beta_j \sigma_j \overline{S}_j + (\mu_j + \gamma_j - \sigma_j)^2 < (\mu_j + \gamma_j + \sigma_j)^2,$$
$$(1-u)\beta_j \overline{S}_j < (\mu_j + \gamma_j).$$

From the latter inequality, we can define the reproduction number,

$$R_0 = \max_{j=1,\ldots,n} \frac{(1-u)\beta_j \overline{S}_j}{\mu_j + \gamma_j}.$$

B Solution of the Optimal Control Problem

The solve (16), we make use of Pontryagin's maximum principe [1–3]. Firstly, we need to formulate the Hamiltonian function of our optimal control problem, given by:

$$H = c_1 P - e^{c_2 u} + \phi_P \dot{P} \sum_{j=1}^{n} \phi_{S_j} \dot{S}_j + \sum_{j=1}^{n} \phi_{E_j} \dot{E}_j + \sum_{j=1}^{n} \phi_{I_j} \dot{I}_j + \sum_{j=1}^{n} \phi_{R_j} \dot{R}_j + \eta u. \tag{17}$$

In the above equation, ϕ_P represents the co-state variable corresponding to the original variable P; similarly, the subscript of the remaining co-state variables $\phi_{\_}$ indicates the corresponding original variable. In addition, $\eta \geq 0$ is a penalty multiplier added to ensure that $u \geq 0$; at optimality we must have $\eta u^* = 0$.

By deriving the Hamiltonian with respect to the original variables in (9)-(13), we obtain adjoint system of equations with respect to the co-state variables:

$$\begin{aligned}
\frac{d\phi_P}{dt} &= -\frac{\partial H}{\partial P} = -\, c_1\\
\frac{d\phi_{S_j}}{dt} &= -\frac{\partial H}{\partial S_j} = (\phi_{S_j} - \phi_{E_j})\,(1-u)\,\beta_j I_j\\
\frac{d\phi_{E_j}}{dt} &= -\frac{\partial H}{\partial E_j} = \sigma_j(\phi_{E_j} - \phi_{I_j})\\
\frac{d\phi_{I_j}}{dt} &= -\frac{\partial H}{\partial I_j} = (\phi_{S_j} - \phi_{E_j})(1-u)\beta_j S_j + \phi_{I_j}(\mu_j + \gamma_j) - \phi_{R_j}\gamma_j + \phi_P\mu_j +\\
&\qquad + \mu_j\Big(\sum_{i=1;i\neq j}^{n} \phi_{S_i}\Big)\\
\frac{d\phi_{R_j}}{dt} &= -\frac{\partial H}{\partial R_j} = \delta_j(\phi_{R_j} - \phi_{S_j})
\end{aligned}$$

with transversality conditions,

$$\phi_P(T) = \phi_{S_j}(T) = \phi_{E_j}(T) = \phi_{I_j}(T) = \phi_{R_j}(T) = 0,\ \forall\, j = 1, \cdots, n.$$

Theorem 3 *The solution to the optimal control problem in* (16) *yields:*

$$u^* = \max\left\{0, \frac{1}{c_2}\ln\left(\frac{1}{c_2}\sum_{j=1}^{n} S_j I_j \beta_j(\phi_{S_j} - \phi_{E_j})\right)\right\}. \tag{18}$$

Proof The optimal solution u^* must satisfy:

$$\frac{\partial H}{\partial u^*} = -c_2 e^{c_2 u^*} + \sum_{j=1}^{n} S_j I_j \beta_j(\phi_{S_j} - \phi_{E_j}) + \eta = 0.$$

Thus, isolating u^*, we obtain,

$$\begin{aligned}
c_2 e^{c_2 u^*} &= \sum_{j=1}^{n} S_j I_j \beta_j(\phi_{S_j} - \phi_{E_j}) + \eta\\
e^{c_2 u^*} &= \frac{\sum_{j=1}^{n} S_j I_j \beta_j(\phi_{S_j} - \phi_{E_j}) + \eta}{c_2}\\
c_2 u^* &= \ln\left(\frac{\sum_{j=1}^{n} S_j I_j \beta_j(\phi_{S_j} - \phi_{E_j}) + \eta}{c_2}\right)\\
u^* &= \frac{1}{c_2}\ln\left(\frac{\sum_{j=1}^{n} S_j I_j \beta_j(\phi_{S_j} - \phi_{E_j}) + \eta}{c_2}\right)
\end{aligned}$$

If $u^* > 0$, we necessarily have $\eta = 0$, since $\eta u^* = 0$. Consequently, the optimal control can be expressed as:

$$u^* = \frac{1}{c_2}\ln\left(\frac{\sum_{j=1}^{n} S_j I_j \beta_j(\phi_{S_j} - \phi_{E_j})}{c_2}\right). \tag{19}$$

But, if $u^* = 0$, then

$$\begin{aligned}
u^* = \frac{1}{c_2}\ln\left(\frac{\sum_{j=1}^{n} S_j I_j \beta_j(\phi_{S_j} - \phi_{E_j}) + \eta}{c_2}\right) &= 0,\\
\ln\left(\frac{\sum_{j=1}^{n} S_j I_j \beta_j(\phi_{S_j} - \phi_{E_j}) + \eta}{c_2}\right) &= 0,
\end{aligned}$$

which, considering the properties of the logarithm function, yields

$$\frac{\sum_{j=1}^{n} S_j I_j \beta_j(\phi_{S_j} - \phi_{E_j}) + \eta}{c_2} = 1,$$

$$\sum_{j=1}^{n} S_j I_j \beta_j(\phi_{S_j} - \phi_{E_j}) + \eta = c_2,$$

$$\eta = c_2 - \sum_{j=1}^{n} S_j I_j \beta_j(\phi_{S_j} - \phi_{E_j}) > 0.$$

The inequality above holds true because, by definition, $\eta u^* = 0$ and $\eta \geq 0$; the case where $\eta = 0$ was already explored in Eq. (19).

Hence, Eq. (20) below summarises the optimal control results:

$$u^* = \max\left\{0, \frac{1}{c_2}\ln\left(\frac{1}{c_2}\sum_{j=1}^{n} S_j I_j \beta_j(\phi_{S_j} - \phi_{E_j})\right)\right\}. \tag{20}$$

References

1. Kirk DE. Optimal Control Theory: An Introduction. Networks Series. Prentice-Hall; 1970.

2. Bryson AE, Ho YC. Applied Optimal Control: Optimization, Estimation, and Control. CRC Press; 1970.

3. Pontryagin LS, Boltyanskie VG, Gamkrelidze RV, Miŝenko EF, Trirogoff KN. The Mathematical Theory of Optimal Processes. In: Neustadt LW, editor. Karreman Mathematics Research Collection. Interscience Publishers; 1962.
